# Current and Future Trends in the Laboratory Diagnosis of Sexually Transmitted Infections

**DOI:** 10.3390/ijerph18031038

**Published:** 2021-01-25

**Authors:** Giorgia Caruso, Anna Giammanco, Roberta Virruso, Teresa Fasciana

**Affiliations:** 1 U.O.C. of Microbiology and Virology, ARNAS “Civico, Di Cristina and Benfratelli”, 90127 Palermo, Italy; 2 Department of Health Promotion, Mother and Child Care, Internal Medicine and Medical Specialties, University of Palermo, Via del Vespro 133, 90127 Palermo, Italy; anna.giammanco@unipa.it (A.G.); teresa.fasciana@virgilio.it (T.F.); 3 U.O.C. of Microbiology, Virology and Parassitology, A.O.U.P. “Paolo Giaccone”, 90127 Palermo, Italy; robivirruso@gmail.com

**Keywords:** sexually transmitted infections, diagnostic, microscopy, culture, immunoassay, NAAT, point-of-care test, biosensing, metagenomics, culturomics

## Abstract

Sexually transmitted infections (STIs) continue to exert a considerable public health and social burden globally, particularly for developing countries. Due to the high prevalence of asymptomatic infections and the limitations of symptom-based (syndromic) diagnosis, confirmation of infection using laboratory tools is essential to choose the most appropriate course of treatment and to screen at-risk groups. Numerous laboratory tests and platforms have been developed for gonorrhea, chlamydia, syphilis, trichomoniasis, genital mycoplasmas, herpesviruses, and human papillomavirus. Point-of-care testing is now a possibility, and microfluidic and high-throughput omics technologies promise to revolutionize the diagnosis of STIs. The scope of this paper is to provide an updated overview of the current laboratory diagnostic tools for these infections, highlighting their advantages, limitations, and point-of-care adaptability. The diagnostic applicability of the latest molecular and biochemical approaches is also discussed.

## 1. Introduction

Sexually transmitted infections (STIs) are some of the most frequent communicable conditions globally. They represent a major health and social issue and, in particular, four treatable STIs contributed to up to 376.4 million new infections in 2016 [1], corresponding to approximately one million new cases per day. This number included infections due to *Chlamydia trachomatis* (CT) (127.2 million), *Neisseria gonorrhoeae* (NG) (86.9 million), *Trichomonas vaginalis* (TV) (156 million) and *Treponema pallidum* subspecies *pallidum* (TP) (6.3 million). Additionally, the World Health Organization (WHO) [2] estimated up to 417 million infections of herpes simplex virus type 2 (HSV-2) and 291 million women infected with human papillomavirus (HPV). Although frequently underdiagnosed, genital mycoplasmal infections are also among the most common STIs, with a prevalence of 1–3% in the sexually active general population for *Mycoplasma genitalium* (MG), and 20–50% and 40–80% in asymptomatic sexually active women for *Mycoplasma hominis* (MH) and *Ureoplasma urealyticum* (UU), respectively [2].

STIs represent a considerable burden for public health, which is difficult to assess because asymptomatic cases are very common. Undetected infections which are not treated can have serious complications, which disproportionately affect women and their newborn babies. Congenital syphilis can cause spontaneous abortion and premature delivery [3], and the WHO estimates that this condition is responsible for more than 300,000 fetal and neonatal deaths, and for an increased risk of premature death for 215,000 infants each year [4]. Furthermore, up to 530,000 cases of cervical cancer and 264,000 associated deaths are caused each year by HPV infections [4]. The two most common bacterial STIs, CT and NG, not only cause pelvic inflammatory disease and chronic pelvic pain in women, but also result in ectopic pregnancies and miscarriages, preterm labor, increased risk of mother-to-child HIV transmission, conjunctivitis, and pneumonia in neonates [5]. Although the evidence is inconclusive, MG and TV infection in pregnancy have also been associated with preterm birth [6]. Neonates can become infected with HSV at birth from an infected mother, with potential involvement of the central nervous system and fatal consequences [7]. Both CT and NG can cause irreparable damage to the fallopian tubes, leading to infertility in women [8], and a possible role of these organisms in the reduction of sperm quality and fertility in males has been suggested [9]. Furthermore, STIs increase the susceptibility to human immunodeficiency virus (HIV) infection and its transmission risk due to augmented viral shedding in genital secretions [10].

In 2016, the WHO launched its global strategy for tackling STIs [4]. One of the main cornerstones includes improved surveillance through the development and implementation of better diagnostic algorithms and tests. In particular, the early diagnosis and identification of asymptomatic carriers (screening) is a prerequisite to efficiently guide treatment and prevention interventions for STIs [4,11]. In recent years, the advent of molecular and mass spectrometry tools revolutionized the clinical microbiology laboratory [12]. In particular, the latest technological advancements paved the way for the development and implementation of point-of-care (POC) testing [13].

The purpose of this review is to discuss the latest advancements in the laboratory diagnosis of the main STIs, in particular: (1) the importance of POC and molecular assays in complementing the traditional syndromic approach for the early diagnosis and the identification of asymptomatic carriers; and (2) the current and future technological developments for STI diagnostics (including omics approaches).

## 2. The Limitations of the Syndromic Management of STIs

The syndromic management of STIs is based on obtaining a presumptive diagnosis via the observation of a set of signs and symptoms (“syndrome”) in the patient [14], without the need for laboratory confirmation of the underlying pathogen. This approach is cheap, it can be used in resource-poor and remote areas, and it allows immediate treatment during the first visit to the clinic or health center without any laboratory trained staff or technically demanding procedures [15]. However, the inconspicuous clinical presentation of STIs affects the applicability of the syndromic approach. These infections are often asymptomatic and the same set of signs and symptoms may correspond to different underlying organisms, leading to subjective judgement. Furthermore, even physiological discharge may be misinterpreted as pathological [15].

The syndromic approach is efficient and cost-effective when most cases are symptomatic, as in the case of CT and NG infections causing urethral discharge in men [16,17]. The opposite situation is seen for vaginal discharge in women, as shown by various studies. For instance, based on the results of a study that collected samples from sex workers in India, Desai and colleagues [18] estimated that a syndromic approach based on vaginal discharge had a low specificity (50–55%) and a very low positive predictive value (PPV) (from 11.5% for CT to 24.6% for TV), missing 30–40% of cases of CT and NG and leading to up to 90% of cases being treated without infection. Similar results have been reported in Delhi [19], where the reported specificity of the syndromic approach using vaginal discharge as a symptom was only 37.5%. Vaginal discharge showed an even lower PPV (14.1%) in pregnant women in Sudan [20].

Hence, the syndromic approach has poor predictive value and should not be used for screening, as it can lead to either overtreatment or undertreatment. In the first case, antibiotics are prescribed to healthy patients, contributing to the development of antimicrobial resistance, unnecessary costs, and emotional upset. In the latter case, no antibiotic is given to patients whose test is positive, potentially increasing the risk of reproductive sequelae and other health complications, in addition to the further spread of STIs among the population [21,22].

## 3. The Need for STI Laboratory Confirmation and the Advantages of Point-Of-Care Testing

The clinical presentation per se does not always allow a specific STI to be identified. In these cases, etiological diagnosis via laboratory confirmation of the underlying organisms is essential. Other than supporting a more rational use of antimicrobials, laboratory confirmation is pivotal in surveillance (to determine the true scale of the spread of STIs in communities) and in screening (i.e., testing of at-risk people without recognized signs or symptoms) [11]. By identifying infected people regardless of their symptoms, both the complications and further transmission of STIs can be reduced [4]. Approximately 30 different pathogens can be transmitted sexually, ranging from different bacterial species, viruses, fungi, and parasites [23], so the choice of the appropriate diagnostic approach can be difficult for physicians.

To target the most relevant pathogens, various tools have been developed for the laboratory diagnosis of STIs throughout the years [11]. The choice of the most appropriate diagnostic test (Box 1) relies not only on the performance of the tool itself in terms of sensitivity, specificity, and predictive values, but also on the logistics (technical requirements, cost, throughput) and its purpose.

Box 1 **Characteristics to consider when choosing a diagnostic assay (adapted from [11]).**

- Sensitivity: ability to correctly identify individuals with the infection (true positive rate)
- Specificity: ability to correctly identify individuals without the infection (true negative rate)
- Positive predictive value (PPV): probability that positive individuals truly have the infection
- Negative predictive value (NPV): probability that negative individuals truly do not have the infection
- Complexity: includes all the technical requirements (equipment, reagents, personnel) needed for optimal test performance
- Cost: both materials- and labour-related
- Throughput: number of tests completed in a given amount of time
- Time to result: time needed to get a response from the test

The use of the most sensitive and specific tests is often impractical in resource-poor or remote areas due to their high cost and technical requirements. In addition, considering the clinical presentation and the severe complications associated with some STIs if left untreated, the “ideal” diagnostic test should be quick so that the patient is treated on the spot. In these settings, it is a common for patients to not return to the clinic for treatment while waiting for a laboratory result [24], and loss to follow-up can also affect the sensitivity of laboratory diagnosis. This is well exemplified by a study undertaken on female sex workers in Benin [25]; the authors found that up to 57.6% of infected women did not return to the clinic within 10 days of the visit, resulting in a decrease in the laboratory test sensitivity from 74.6% to 28.2%.

Point-of-care (POC) tests represent an answer to the problem of needing to reach a diagnosis quickly outside of a standard laboratory. By definition, a POC test can be performed at the patient’s hospital bedside or own house, the physician’s office, or in the field [26]. Ideally, a POC test should meet the WHO affordable, sensitive, specific, user-friendly, rapid and robust, equipment-free and deliverable to end-users (ASSURED) criteria [27]. 

POC tests based on the detection of pathogen nucleic acids (NAs), antigens, or even antibodies have been developed for a variety of infectious diseases [13,28]. Regarding STIs, POC tests are available for CT [29], NG [30], and TV [31], for example. Some of these POCs, such as the OSOM^®^ antigen test for TV, can be executed directly by patients with comparable sensitivity and specificity to that of a test performed by a clinician in a hospital setting [32], and patients can easily collect the specimen and interpret the result at home [33]. The development and implementation of POCs for STI management is considered a priority by WHO [4], and target product profiles (TPPs) for POCs have been developed [34,35].

Recent systematic reviews have shown that, in many instances, the use of POCs can result in a substantial reduction in both missed treatments and overtreatment [28,36]. Treatment uptake is also improved, and patients show appreciation for receiving a quick and specific diagnosis, which also facilitates the treatment of the partners of patients [37].

POC testing is so widespread that it also has an important role in the management of bacterial vaginosis (BV), even though it is a dysbiotic condition. However, BV is the most common vaginal disorder [38], affecting approximately 30% of women of reproductive age and up to 50% in sub-Saharan Africa [39,40]. Notwithstanding, BV results from a dysbiosis, and the transmissibility of BV implies that it may also result from external contamination in addition to internal imbalance, resulting in a true STI [41,42]. The major issue with BV is that its diagnosis remains problematic and subjective; hence, innovations in this field are urgently needed, and reliable and low-complexity tests are required.

A POC test detecting sialidase activity in vaginal discharge has been implemented, with an excellent sensitivity and specificity in comparison with standard Gram staining [43]. Metabolomics-based POC tests based on the identification of characteristic organic compounds (i.e., biogenic amines) are being developed, such as the VGTest, which measures the presence of these molecules using a portable desktop ion mobility spectrometer (IMS) [44,45]. Given the potentially relevant adverse effect on women’s reproductive health, promotion of the implementation of more rapid and accurate POC tests to improve the routine diagnosis of BV should be a primary issue.

## 4. Overview and Latest Developments in the Laboratory Diagnosis of STIs

Currently available laboratory diagnostic approaches for the detection of active STIs are summarized in Table 1 and discussed in more detail below. The following sections focus on POC formats, molecular diagnostics, and the latest technological advancements.

### 4.1. Microscopy and Culture

Most of the etiological agents of STIs can be visualized microscopically or grown under laboratory conditions following isolation from clinical samples. These approaches are still in use (particularly for bacteria) in resource-poor settings and in peripheral or intermediate-level laboratories, but their performance varies. Both microscopy and culture are ideal for confirming the patient diagnosis and identifying a proper course of treatment.

Microscopy is cheap and does not require special equipment, and can be performed at the point-of-care if needed [11]. However, sensitivity is affected by the microscopist’s skills and tends to be lower in asymptomatic patients, as seen for example in gonorrhea [30] and trichomoniasis [46]. Another drawback is that microscopic analysis should be carried out within 10 min of sample collection, making the examination unsuitable for high throughput laboratories [47]. 

Culturing the organisms using specific media shows good sensitivity and specificity, and is a highly standardized method. Despite being a slow process (often taking several days depending on the organism), it remains the only reliable method to test for antimicrobial resistance. The efficiency and precision of culture has been recently increased due to the use of matrix-assisted laser desorption/ionization–time-of-flight (MALDI-TOF) mass spectrometry [48]. This technique can precisely identify not only the species, but also the subspecies or serotype of the organism within minutes. Despite its ability to identify some viral species, spectrometry still does not enable the routine identification of viruses due to a lack of commercially available databases [48].

Some drawbacks of the culture approach include the fact that organisms such as TP [49] or HPV [50] are not culturable, and the growth of CT [29] or HSVs [51] requires specialized laboratories and is both time-consuming and expensive. In addition, NG is a fragile microorganism, and hence culture assays may exhibit low sensitivity due to inappropriate transportation conditions [52].

Furthermore, cultural approaches could miss some fastidious microorganisms with particular growth requirements that need the organism to be viable, making the sample collection step and storage extremely important to ensure the reliability of the result.

### 4.2. Immunological Assays 

Immunoassays detect either the pathogen’s antigens or the presence of antibodies produced by the immunological response to infection. These tools can be adapted into POC formats, particularly in the form of lateral flow immunochromatographic (ICT) assays. However, they are not readily available for all STIs (for example, genital mycoplasmas and HPV), and only a few of these tests have been thoroughly validated in clinical settings.

Antigen detection-based assays allow the diagnosis of current infections. A few of these tests have been developed and tested for the detection of NG, showing relatively high specificity and returning results in just 25–40 min [53]. However, their sensitivity is highly variable and, in many instances, too low [30]. The same issues apply to antigen tests for the detection of CT [29], and the WHO does not recommend their use if other methods are available [11]. The reduced sensitivity represents a problem, particularly in swabs collected from asymptomatic cases [11,29,51]. Furthermore, because the interpretation is visual, the result is operator-dependent and this fact may impair diagnosis, particularly in weakly positive cases. However, the development of automated strip readers and the use of artificial intelligence can help to manage this disadvantage [54].

A few antigen rapid assays have been developed for trichomoniasis [46]. The OSOM^®^ Trichomonas Rapid Test is a good example of an efficient POC test [31]. Featuring good sensitivity and specificity, this POC test can be conducted at home and was found to be highly acceptable by women [31]. Despite their sub-optimal sensitivity, antigen-based POC tests can improve patient management (leading to more cases being treated) in high infection prevalence contexts due to their rapidity. This has been observed for both CT [24] and TV [55].

Antibody tests have the drawback of detecting past exposure to the pathogen, and further tests are needed to confirm the presence of a currently active infection [11,29,51]. However, serological assays for the presence of serum antibodies represent a sensitive and specific tool for the diagnosis of syphilis [49]. A variety of serological assays have been developed for this infection and they play a crucial role in screening pregnant women to prevent adverse pregnancy outcomes. These tests show a good performance in both sensitivity (90% or more depending on the test) and specificity (>95%), and their ICT format makes them ideal for resource-poor settings [56]. Tests are now also available to detect both treponemal and non-treponemal antibodies, allowing discrimination between past infection and active syphilis [56].

Although the multiplexing potential of immunoassay tests is still in its infancy, some examples are available. Latest technological developments regarding microfluidics and biosensors have sparked the development of a test for the parallel detection of CT and NG [57]. The assay is sensitive and rapid, and can be used successfully on crude urine [57]. Simultaneous antibody detection of both TP and HIV can be now accomplished through various ICTs with excellent sensitivity and specificity for both pathogens [56]. Interestingly, a duplex enzyme-linked immunosorbent assay (ELISA) for the detection of HIV/syphilis antibodies in fingerprick blood has been adapted in a device which is operated by a smartphone, facilitating the testing and reading of the results directly in the field [58]. The combination of nanotechnology and immunoassays has proved to be a promising approach, with the introduction of quantum dots (fluorescent labels of protein size) for labelling secondary antibodies, replacing conventional fluorescent molecules [59]. The use of quantum dots can also increase the potential for multiplexing in immunoassays [60].

### 4.3. Nucleic Acid Amplification Tests (NAATs) and Detection

Methods based on the amplification and detection of an organisms’ nucleic acids (DNA or RNA) in clinical samples now represent the gold standard for the diagnosis of many STIs. Nucleic acid amplification tests (NAATs) are particularly important for organisms for which traditional tools have always been either unavailable or too expensive, such as *C. trachomatis*, genital mycoplasmas, and viruses. Overall, these tools overcome some important limitations of microscopy and culture-based approaches.

NAATs have a significantly lower turnaround time (they can be easily automated, and the time to result is significantly lower than that of traditional approaches), they allow the detection of organisms which cannot be grown in culture, and they have a significantly higher sensitivity (a dramatically relevant advantage for asymptomatic infections where the organisms’ load is low) [61,62]. Due to their high sensitivity, NAATs are an invaluable tool for detecting extragenital infections, as shown in the case of rectal or pharyngeal gonorrhea in men [63]. Furthermore, NAATs do not require viable organisms, allowing the collection and utilization of more diverse and less invasive biological samples (such as urine or vaginal swabs) which can be easily self-collected; they can be automated and standardized, and they can be performed by minimally trained people; and they can be multiplexed, allowing the detection of multiple organisms from the same sample and in a single test run [64,65]. Due to the latest technological advances, NAATs also have significant potential to become the POC tests of the future for STIs due to a reduction in their cost [36].

#### 4.3.1. PCR-Based Assays

Polymerase chain reaction (PCR) has revolutionized clinical microbiology. Tiny amounts of DNA or RNA can be amplified from samples which are unsuitable for analysis by traditional methods; thus, it is not surprising that PCR-based tools now play a central role in the laboratory diagnosis of infectious diseases. Various PCR-based assays (single-step, nested, real-time, singleplex, or multiplex) have been developed for STIs, thereby simplifying the diagnostic workflow and reducing the turnaround time. Many of these tests have been perfected and are now commercially available; multiplex assays are available for the detection of up to eighteen different organisms (e.g., the CLART^®^ STI A&B, Genomica S.A.U., Madrid, Spain), and their sensitivity and specificity are comparable with their respective singleplex assays. Multiplexing allows for the cost-effective analysis of one sample through the exploitation of a syndrome-associated specific panel, hence with an augmented probability of identifying the responsible pathogen.

For instance, the BD Max^™^ CT/GC/TV (MAX) assay (BD Diagnostics, Franklin Lakes, NJ, USA) is a multiplex real-time assay capable of detecting CT, NG, and TV. The test shows an excellent performance using both vaginal and endocervical swabs and male urine, with sensitivities in the range of 91.1–99.1%, and consistently high specificity (≥98.6%) for all the organisms tested [66]. Another example is the Anyplex^™^ II STI-7 (Seegene Inc, Seoul, South Korea), which is based on the same principle, but it can detect up to seven STIs including CT, GC, TV, MG, MH, and two *Ureaplasma* sp. (*urealyticum* and *parvum*). In various studies, including both symptomatic patients and asymptomatic volunteers, the assay performed extremely well in terms of both sensitivity and specificity (≥99%), and showed good concordance and reduced cost compared to similar (but only duplex) platforms [67,68]. The assay was used successfully in Ghana to test asymptomatic women, revealing a high prevalence of MG and co-infections with up to three organisms [69], and confirming the applicability of this assay for the extensive screening of genital mycoplasmas as reported elsewhere [70]. Another multiplex assay, the FilmArray^®^ (BioFire Diagnostics, Salt Lake City, UT, USA), can detect up to nine STIs (CT, GC, TV, MG, and UU, plus TP, *Haemophilus ducreyi*, and HSV 1 and 2) [71].

Several PCR-based assays have been developed specifically for the detection of *T. pallidum*, not only from genital swabs and lesions, but also from blood and cerebrospinal fluid [74], and even semen [75]. Although they show high sensitivity and specificity, these tests are not commercially available and their use is limited to some laboratories [49]. There are various PCR-based assays for the detection of viral STIs, such as HSV1,2 [51] and HPV [76], many of which are commercially available and approved by the relevant regulatory bodies, and which constitute the tests of choice for these infections.

There are some PCR-based platforms which have potential as POCs. The GeneXpert^®^ (Cepheid, Sunnyvale, CA, USA) platform is a real-time PCR benchtop instrument adaptable for the detection of a variety of infections. In particular, the CT/NG assay running on this platform showed excellent sensitivity (100%) and specificity (≥99.8%) in detecting these organisms in the urine of both men and women [77]. However, its high price and the need for electricity do not allow this platform to be considered as a fully realized POC test [26].

Current PCR-based assays have reached extremely high levels of sensitivity and specificity of detection, and they are powerful tools for screening samples for multiple organisms. However, their ability to be implemented as POC tests in resource-poor settings is still hindered by their relatively high cost and the need for specialized instrumentation (thermocycler) and laboratories.

#### 4.3.2. Isothermal Amplification Assays and Microfluidics

Isothermal amplification (IA) assays can offer a rapid and cost-effective solution for the diagnosis of STIs compared to PCR-based assays. In IA assays, the amplification of the target DNA/RNA does not require thermal cycling (as in PCR) and happens at a significantly lower and constant temperature; the time-to-result is generally reduced (<1 h), and the amplification results can be easily assessed visually (by observing turbidity or a change of color in the reaction tube) [78]. There are many IA methods which have been exploited in molecular diagnostics involving different chemistries. IA approaches used for STIs include loop-mediated isothermal amplification (LAMP), transcription-mediated amplification (TMA), strand displacement amplification (SDA), helicase-dependent amplification (HDA), and recombinase polymerase amplification (RPA).

The enzymes used in IA assays are less affected by the inhibitory substances present in biological samples, opening the possibility of reducing both the time and cost associated with sample preparation and extraction. A LAMP assay developed for the detection of NG showed the same sensitivity of a reference PCR test when used on crude urine [79]. The applicability of using samples without performing traditional DNA extraction was also proven for a LAMP assay used to detect CT in endocervical swabs, with a lysis step of just five minutes before amplification [80]. 

Target amplification can be assessed using a lateral flow approach in the form of paperfluidic devices, as shown in a similar semi-quantitative LAMP-based approach for CT [81]. Such a format is used in rapid diagnostic tests, such as the HDA-based AmpliVue *Trichomonas* assay (Quidel, San Diego, CA, USA) for TV [82].

IA approaches, and in particular RPA, can produce results very rapidly. Notable examples are the assay for the detection of CT directly from urine, giving a result within 20 min [83], or the 15-min time-to-result of a prototype for the simultaneous detection of CT and NG [84]. In addition, the TMA approach (which targets pathogen RNA rather than DNA) has been exploited for STI testing, most notably in the form of the Aptima^®^ platform (Hologic, Marlborough, MA, USA), which has been used to detect NG/CT and TV [46].

Multiplexing is also possible with IA approaches, and recent advances in microfluidics technology are pushing the boundaries of the “lab-on-a-chip” idea, in which sample extraction, amplification, and detection occur sequentially in the same instrument. Considerable promise has been shown by microfluidics. This technology is characterized by a precisely engineered manipulation of small volumes of fluids in channels on the micrometer scale. Microfluidics use ranges from nucleic acid purification to immunoassays, and its use suggests a revolution of medical diagnostics to overcome existing limitations. It allows for faster assay time-to-result, lower technical requirements for laboratories, smaller volumes of reagents, and increased automation and integration levels [85]. Furthermore, complex technologies can be adapted in POC tests through miniaturization. For instance, magnetic bead-based mechanisms allow for the analysis of complex and crude biological samples [86,87]. Real-time amplification is then carried out through miniaturized modules combining thermal cycling and detection. Melting profiles can be used to differentiate PCR products, in addition to melting temperatures, thereby allowing the detection of multiple pathogens simultaneously.

The detection of major causes of STIs is one of the most promising applications of microfluidics [88]. Magnetofluidic cartridges may be assembled, combining reagents for both nucleic acid extraction and amplification with complete process integration from sample to detection, as shown in the case of CT detection operated by a mobile phone interface [89].

A LAMP assay was recently developed using magnetic beads and a capillary-based architecture to simultaneously detect NG and CT [90], and another has been tested for identifying genital mycoplasmas (MG, MH, UU) [91], showing concordance with the results of PCR on clinical samples but with a higher sensitivity at lower DNA concentrations. Another assay exploiting LAMP applied to the concept of “lab-on-a-chip” successfully detected NG, CT, MH, and UU directly from genitourinary secretions [92]. The Illumigene (Meridian Bioscience, Cincinnati, OH, USA) HSV-1,2 multiplex LAMP assay has been thoroughly evaluated in a multicenter study, and detects and differentiates the two viruses with a higher sensitivity compared to other molecular assays and even higher than that of culture [93]. A very rapid detection (less than one hour) has also been successfully achieved simultaneously for CT and NG using a strand invasion-based (SIBA) approach [94].

#### 4.3.3. Sensitive Detection without Amplification

Regardless of the approach (PCR-based or IA), NAATs require a strategy to amplify DNA/RNA from clinical samples. Novel methods have been described for the detection of DNA or RNA directly from the sample without amplification. These technologies highlight the possibility of skipping the amplification steps, potentially resulting in a significant reduction in sample handling and processing times, and making POC testing possible.

One of these approaches, called microwave-accelerated metal-enhanced fluorescence (MAMEF), combines low-power microwave heating to release DNA directly from a sample and metal-enhanced fluorescence for its detection. This method was first described for the rapid detection (less than a minute) of cultured CT [95]. In another study, MAMEF was used to detect CT and plasmid DNA from genital swabs within nine minutes [96]; despite the suboptimal sensitivity (<90%), the method showed a good agreement with PCR and showed promise to be adapted into a POC test following further improvements.

Much attention is now being given to the use of nucleic acid-binding proteins as biosensors for diagnostics [97]. These include zinc-finger (ZF) and transcription activator-like effector (TALE) protein domains, and the clustered regularly interspaced short palindromic repeat (CRISPR)-derived RNA-guided engineered Cas proteins. These programmable proteins can be used to detect very specific DNA/RNA sequences previously amplified via PCR or IA, but their nucleic acid binding properties also allow detection without amplification. Although the use of ZF and TALE proteins has not been closely explored in molecular diagnostics [97], Cas proteins (in particular the Cas12 class of molecules) have attracted increased attention for the POC detection of many pathogens [98].

The use of Cas12 proteins has been recently described for viral STIs. Extremely low amounts of HPV DNA (in the attomolar range) were detected using a Cas12 system following preliminary RPA amplification [99]. Furthermore, the assay can discriminate two HPV types (16 and 18) from both anal swabs and cell cultures. A similar strategy was implemented to detect HPV16/18 from swabs and human plasma using a 3D-printed microfluidic device [100]. Electrochemical biosensors for the detection of HPV DNA without amplification via Cas12 have also been reported [101]. Interestingly, a method for the detection of *Mycoplasma* DNA (to detect culture contamination) using RPA and Cas12 with a naked eye in-tube visual readout has also been described [102]. It is expected that more assays for the diagnosis of STIs will appear in the future using programmable nucleic acid binding proteins and exploiting POC platforms.

#### 4.3.4. Detecting Antimicrobial Resistance 

In addition to the detection of the organism per se, it is important to identify the presence of potential antimicrobial resistance (AMR). This information is pivotal, not only to guide the most appropriate pharmacological treatment course for the patient, but also to prevent the further selection and spread of resistance determinants. AMR resistance is a well-known issue for NG and MG globally, but it is also emerging in CT, TP, and TV [103]. Currently validated methods for the detection of AMR in clinical isolates are time-consuming and involve the cultivation of the organisms followed by in vitro antimicrobial susceptibility testing [104].

If the molecular mechanisms of resistance are known, NAATs can be used for the rapid (and potentially POC) detection of resistance genes and mutations. PCR-based molecular assays for this purpose have been developed for STIs, including tests for the detection of macrolide resistance in MG [105] and fluoroquinolone resistance in NG [106]. The absence of known AMR determinants for some antibiotic classes (e.g., fluoroquinolones) has high positive predictive value for phenotypic susceptibility. Conversely, other antimicrobial classes, such as cephalosporins, penicillins, and macrolides, are so complicated that an accurate prediction of resistance cannot be performed due to multiple underlying mechanisms of resistance [107]. Multiplex molecular tests can provide a rapid tool to detect and monitor the prevalence of resistance genes and mutation in at-risk populations [108], and their adaptation to POC formats would benefit patient treatment and management. However, AMR is a complex phenomenon involving several molecular mechanisms, and novel resistance genes and mutations constantly emerge, so the clinical relevance of nucleic acid-based tools remains to be verified against traditional phenotypic tools [107].

## 5. The New Possibilities Of-Omics

In recent years, DNA and RNA sequencing technologies have been extensively used to characterize the microbial communities—usually indicated as the microbiome—inhabiting the human body. Metagenomics allows the characterization of the collective genome of microbial communities in a sample, leading to the identification of novel organisms without the need for culture. Furthermore, changes in microbial communities which are potentially associated with disease can be studied. A dysbiotic vaginal environment, like the one observed in bacterial vaginosis (BV), is interlinked with an augmented risk of contracting STIs [109] and adverse pregnancy outcomes [6].

Metagenomic approaches allow for the identification of specific bacterial species, such as *Gardnerella vaginalis* and *Atopobium vaginae*, which are predictive for BV [110]. The presence of these two bacteria has also been associated with preterm birth [111]. Next generation sequencing has also been used to detect AMR genes directly from urine samples in complicated urinary tract infections [112], potentially paving the way for rapid POC nanopore-based whole genome sequencing technologies. Potentially, these approaches could assist in identifying pathogens in culture-negative clinical samples, as recently shown in the case of synovial fluid [113] and urine [114]. However, before metagenomic approaches can become part of the routine clinical diagnosis of STIs, additional aspects must be considered. Among others, these include developing appropriate laboratory workflows and standards to avoid contamination and difficulty in the interpretation of the results, reducing the cost and turnaround time of the instrumentation, and establishing appropriate (and possibly more user-friendly) bioinformatic analysis pipelines [115].

Culturomics is another promising approach for its potential applications to STIs. It consists of the high-throughput analysis of microbes cultured under different conditions using MALDI-TOF mass spectrometry [116]. Culturomics often integrates the information obtained through metagenomics, and allows the functional analysis of enzymes and the metabolic activity of the cultured microbes to further refine the culture conditions. Despite still being in its infancy for STIs, culturomics can aid in the characterization of bacterial species putatively associated with BV [117].

## 6. Conclusions

Due to the limitations of a syndromic approach, the laboratory diagnosis of STIs is essential to ensure timely and appropriate patient treatment. Furthermore, the actual burden and spread of these infections can only be quantified by including the asymptomatic carriers. Tools based on antigen and DNA/RNA detection have revolutionized the field, allowing for a more rapid and sensitive diagnosis compared to traditional microscopy or culture, and highlighting the possibility of STI screening in at-risk groups. Advancements in the development of novel materials, chemistries, and portable devices has created the potential for POC testing.

Some challenges remain for STI diagnostics. NAATs are a powerful tool, but further improvements are needed to make them less technologically demanding so that they may be more affordable in resource-poor settings. Isothermal tools and their future refinements show great promise in this respect, particularly regarding pathogen detection with minimal sample handling and without the need for amplification due to protein-based biosensors. Rapid testing for the presence of antimicrobial resistance remains an important issue for STIs. Long-term goals include thorough research to improve the development of microfluidic technologies and their definitive establishment in diagnostic platforms. Thus, independence from cold chain transport and storage, in addition to a further reduction in the workflow, can be achieved, encouraging the spread of laboratory diagnostics in resource-limited settings and welcoming a new era in healthcare.

Furthermore, the extensive use of next generation sequencing, metagenomics, and culturomics on clinical samples could provide assistance, not only in the characterization and detection of AMR mechanisms, but also in developing strategies for previously unculturable organisms. Whole-genome sequencing can be used to track the origin and evolution of hospital outbreaks [115], and allows for the high-resolution typing of microorganisms such as NG to unravel the sexual networks behind STI transmission [118]. Untargeted metagenomic sequencing from a clinical sample can, in principle, identify any microorganism present without previous knowledge of its genome. Appropriate databases and bioinformatic pipelines allow for the rapid identification of pathogens [119], and metagenomics can be used to discover novel viruses in animal and human samples [120]. The use of these approaches on genito-urinary samples could lead to a better understanding of the microbial communities and their impact on the physiology and pathology of the genito-urinary tract, and to discover potentially new pathogens. The widespread and routine use of -omics technologies in a clinical context remains to be seen, particularly in laboratories with limited economic and technical resources, but the future nevertheless appears promising.

Overall, novel technological solutions should be focused on improving the sensitivity, specificity, and cost of current POC tests. Cheap and user-friendly tests for STIs could be routinely used on a much larger scale, resulting in a significant reduction in long-term morbidity and also in costs for the healthcare system. Significant advances have been made in POC testing for STIs, and more tests are in the pipeline [121]. However, the need remains for better integration of STI POC testing into healthcare systems [122]. Due to the hidden nature of STIs, ensuring the extensive and rapid screening of at-risk people and their partners is pivotal to successfully controlling these infections.

## Figures and Tables

**Table 1:** Comparison of current laboratory diagnostic tools for sexually transmitted infections (STIs) based on performance, applicability, and adaptability to a point-of-care (POC) format.

Organism	Method	Sample Type(s)	Sensitivity	Specificity	Technical Complexity/Cost	Throughput/Automation	Multiplexing	Uses	POC Potential	References
*N. gonorrhoeae*	Microscopy (Gram-stained smears)	Swabs (EC, UR, CO)	Low for women and ASYM men; high for SYM men	Low for women and ASYM men; high for SYM men.	Low/low	Moderate/no	No	Diagnosis	Yes	[11,17,30,53]
	Culture (selective media)	Swabs (EC, UR, CO, OP, VA, RE)	Moderate–high	Very high	Moderate/moderate	Moderate/no	No	Diagnosis, AMR testing	No	
	Antigen detection (OI, ICT)	Swabs (EC, CE, VA), urine	Low–moderate	High	Low–moderate/moderate	Moderate/no	Yes	Diagnosis, screening (potential)	Yes	
	NAAT (PCR, IA)	Swabs (EC, UR, CO, OP, VA, RE), urine	Very high	Moderate–very high	Low for IA; high for PCR	High/possible	Yes	Diagnosis, screening, AMR testing (potential)	Yes	
*C. trachomatis*	Microscopy (DFA)	Swabs (EC, UR, CO, OP, RE)	Low	High	Moderate/low	Moderate/no	No	Diagnosis (recommended for CO swabs)	No	[5,11,29,57]
	Culture (mammalian cells)	Swabs (EC, UR, CO, OP, RE)	Moderate–high	Very high	High/moderate	Low/no	No	Diagnosis, AMR testing, genotyping	No	
	Antigen detection (OI, ICT, biosensors)	Swabs (EC, VA, UR), urine	Low–moderate	Very high	Low–moderate (both)	Low/no	Yes	Diagnosis, screening (potential)	Yes	
	NAAT (PCR, IA)	Swabs (EC, UR, CO, OP, VA, RE), urine, liquid cytology medium	Very high	Very high	Low for IA; high for PCR	High/possible	Yes	Diagnosis, screening	Yes	
*T. pallidum* subsp. *pallidum*	Microscopy (immunohistochemistry, DFA, DF)	Lesions, exudates	Low for DF; high for DFA	Low for DF; high for DFA	Moderate/low	Moderate/no	No	Diagnosis	Possible (only DF)	[11,49,56,57]
	Immunoassays (ICT)	Whole blood, serum, plasma	Moderate–high	High	Low/moderate	Low/no	No	Diagnosis, screening	Yes	
	NAAT (PCR, IA)	Swabs, lesions, blood, cerebrospinal fluid, semen	Highly variable	High	Low for IA; high for PCR	High/possible	Yes	Diagnosis, screening	Possible	
*T. vaginalis*	Microscopy (wet preparations)	Swabs (VA, UR), urine (males)	Low–high for SYM women	Very high	Moderate/low	Low/no	No	Diagnosis	Possible	[11,31,46,55,72]
	Culture (selective media)	Swabs (VA, UR), urine (males)	Moderate–high for SYM women	Very high	Moderate/moderate	Low/no	No	Diagnosis, AMR testing, genotyping	No	
	Antigen detection (ICT)	Vaginal swab	High	Very high	Low/moderate	Low/no	No	Diagnosis, screening	Yes	
	NAAT (PCR, IA)	Swabs (EC, UR, CO, OP, VA, RE), urine	Very high	Very high	Low for IA; high for PCR	High/possible	Yes	Diagnosis, screening	Yes	
*M. genitalium*, *M. hominis*, *U. urealyticum*	NAAT (PCR, IA)	Swabs (EC, VA, UR), urine	Very high	Very high	Low for IA; high for PCR	High/possible	Yes	Diagnosis, screening	Possible	[11,73]
HSV-1,2	Microscopy (Tzanck/Papanicolaou/Romanovsky/ immunoperoxidase staining, DFA)	Skin/mucosal lesions, conjunctival/corneal smears, biopsies, base of vesicles/vesicular fluid smears, tissue sections, swabs	Low for ASYM stains; moderate for DFA	Low for most stains; high for immunoperoxidase staining/DFA	Low–moderate/low	Moderate/low	No	Diagnosis, genotyping (only immunoperoxidase staining/DFA)	No	[11,51]
	Culture (mammalian cells)	Swab, skin lesions, fluid/exudate from vesicle base, mucosal sample without lesions, biopsies, conjunctival/corneal smear	Low–high (depending on the clinical context)	High	High/high	Low/no	No	Diagnosis, genotyping	No	
	Antigen detection (ELISA, ICT)	Swab, vesicular fluid, or exudate from base of vesicle	High for fluid/exudate; low for swabs	High	Low–moderate/moderate	Low/no	No	Diagnosis, screening	Yes	
	NAAT (PCR, IA)	Swab, skin lesions, fluid/exudate from vesicle base, mucosal sample without lesions, aqueous/ vitreous humor, corticospinal fluid, blood	Very high	Very high	Moderate–high	High/possible	Yes	Diagnosis, screening, genotyping	Possible	
HPV	NAAT (PCR, IA)	Swabs (EC), scrapes, tissue biopsies	Very high	Very high	Moderate–high (both)	High/possible	Yes	Diagnosis, screening, genotyping	Possible	[11,56]

HSV = herpesvirus; HPV = human papillomavirus; DFA = direct immunofluorescence assay; DF = dark field; OI = optical immunoassay; ELISA = enzyme-linked immunosorbent assay; ICT = immunochromatographic test; NAAT = nucleic acid amplification test; PCR = polymerase chain reaction; IA = isothermal amplification; AMR = antimicrobial resistance; POC = point-of-care; EC = endocervical; VA = vaginal; UR = urethral; CO = conjunctival; OP = oropharyngeal; RE = rectal; ASYM = asymptomatic; SYM = symptomatic.

## Data Availability

Data sharing not applicable.
